# Ferroquine, the next generation antimalarial drug, has antitumor activity

**DOI:** 10.1038/s41598-017-16154-2

**Published:** 2017-11-21

**Authors:** Artem Kondratskyi, Kateryna Kondratska, Fabien Vanden Abeele, Dmitri Gordienko, Charlotte Dubois, Robert-Allain Toillon, Christian Slomianny, Sébastien Lemière, Philippe Delcourt, Etienne Dewailly, Roman Skryma, Christophe Biot, Natalia Prevarskaya

**Affiliations:** 1 Inserm, U-1003, Laboratory of Excellence, Ion Channels Science and Therapeutics, SIRIC ONCOLille, Université Lille 1, Villeneuve d’Ascq, France; 2 Inserm U908, Université Lille 1, Villeneuve d’Ascq, France; 3 Univ. Lille Nord de France, EA 4515 - LGCgE - Université Lille 1, Cité scientifique, SN3, F-59655 Villeneuve d’Ascq, France; 4 Univ. Lille 1, UGSF, UMR 8576 CNRS, 59650 Villeneuve d’Ascq, France; 5 Present Address: Laboratory of Angiogenesis and Vascular Metabolism, Vesalius Research Center, Department of Oncology (KU Leuven) and Vesalius Research Center (VIB), Campus Gasthuisberg O&N4, Herestraat 49 - 912, B-3000 Leuven, Belgium

## Abstract

Despite the tremendous progress in medicine, cancer remains one of the most serious global health problems awaiting new effective therapies. Here we present ferroquine (FQ), the next generation antimalarial drug, as a promising candidate for repositioning as cancer therapeutics. We report that FQ potently inhibits autophagy, perturbs lysosomal function and impairs prostate tumor growth *in vivo*. We demonstrate that FQ negatively regulates Akt kinase and hypoxia-inducible factor-1α (HIF-1α) and is particularly effective in starved and hypoxic conditions frequently observed in advanced solid cancers. FQ enhances the anticancer activity of several chemotherapeutics suggesting its potential application as an adjuvant to existing anticancer therapy. Alike its parent compound chloroquine (CQ), FQ accumulates within and deacidifies lysosomes. Further, FQ induces lysosomal membrane permeabilization, mitochondrial depolarization and caspase-independent cancer cell death. Overall, our work identifies ferroquine as a promising new drug with a potent anticancer activity.

## Introduction

Despite the tremendous progress in medicine, cancer remains one of the most serious global health problems^1^. One of the reasons of ineffective treatments is conventional therapy resistance arising from genetic alterations in cancer cells and heavily influenced by tumor microenvironment^2^. Indeed, cancer cells habituated to harsh environment, frequently observed in solid tumors, represent the most resistant and dangerous tumor components often responsible for tumor relapse, development and metastases. These cancer cells commonly harbor mutations in tumor suppressor and pro-apoptotic genes (e.g. p53, PTEN, Bax) leading to enhanced apoptosis resistance and aggressiveness^3^. Therefore, elaboration of novel anticancer therapies effectively targeting highly resistant cancer cells is urgently needed.

Inhibition of autophagic-lysosomal function has recently emerged as a promising strategy in cancer therapy. Autophagy, a cellular process in which cells degrade and recycle their own constituents, promotes survival of tumor cells that are under metabolic or therapeutic stresses^4^. Consistent with this, autophagy is upregulated in many cancers and contributes to chemotherapy resistance of tumor cells^5^. Lysosomes, which are central to autophagy, have been established to play a critical role in a number of other fundamental cellular processes, such as secretion and macromolecule recycling^6^. Cancer cells, in particular on the advanced stage, have been reported to be highly dependent on effective lysosomal function influencing invasive growth, resistance to apoptosis and angiogenesis^7^. Moreover, numerous cancer cell types tend to have more fragile lysosomal membranes than normal cells showing the potential for selective targeting^8^. Naturally, inhibition of autophagic-lysosomal function in cancer cells is now considered as a promising direction for the elaboration of efficient anticancer drugs.

Out of different classes of compounds targeting autophagic-lysosomal function in cancer, two antimalarial drugs chloroquine (CQ) and hydroxychloroquine (HCQ) are currently being investigated in clinical trials for cancer therapy^9^. Following the success of CQ and HCQ, a number of other existing antimalarials as well as novel synthetic analogs of CQ have been tested for repurposing as cancer therapeutics^10–12^. Although many of these drugs have shown anticancer properties *in vitro*, just a few molecules have been reported to have single-agent antitumor activity *in vivo*. Moreover, significant toxicity at high doses indicates that there is a need in development of more efficient drugs targeting autophagic-lysosomal function in cancer^13^.

**Figure 1:**
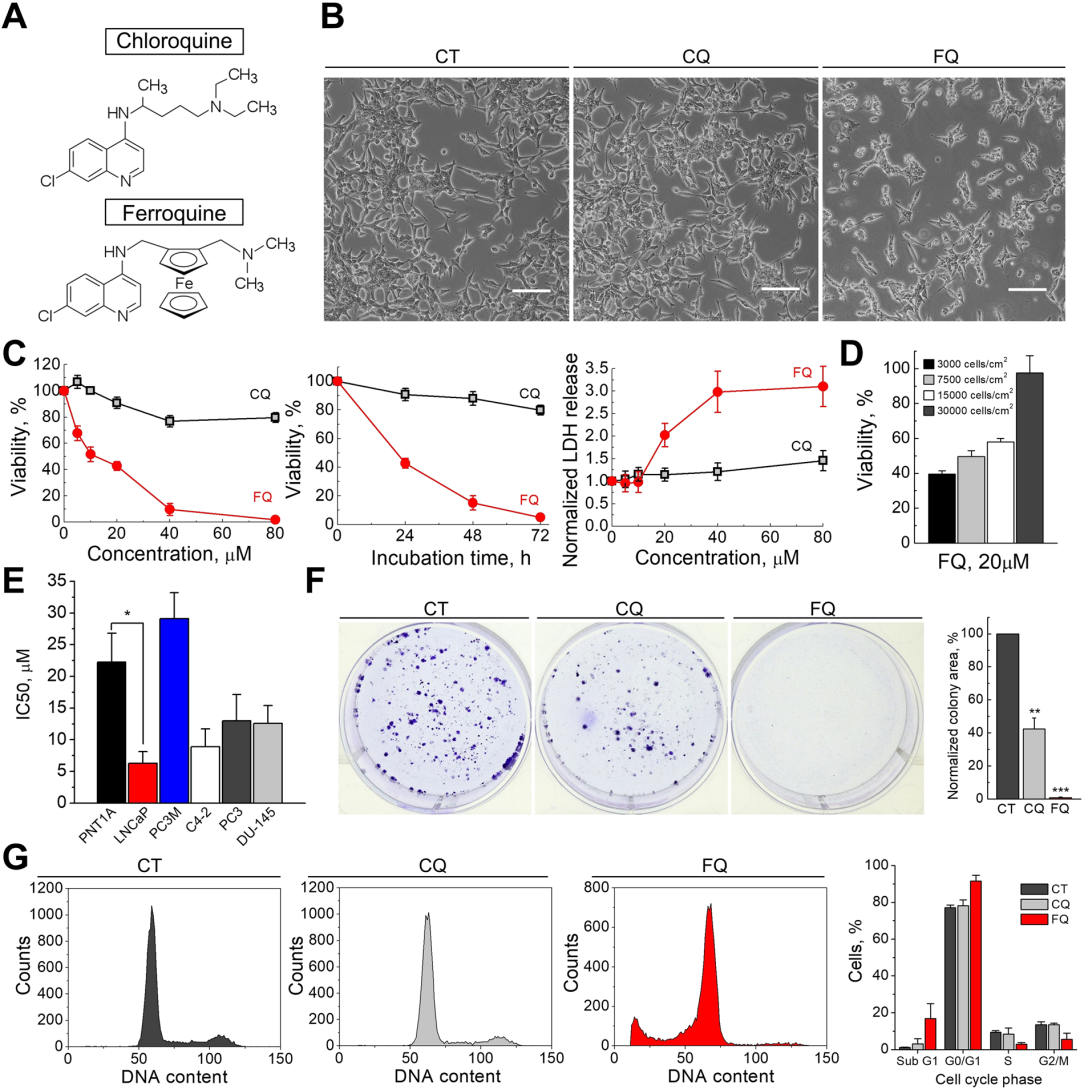
Ferroquine induces proliferative arrest and cancer cell death. (**A**) Chemical structure of CQ and FQ. (**B**) Live imaging of LNCaP cells treated with vehicle, CQ (15 μM) or FQ (15 μM) for 24 h. Scale bars, 100 μm. **(C)** Dose-response of FQ and CQ in viability (MTS, n = 6) and cytotoxicity (LDH, n = 3) assays on LNCaP cells (24 h). (**D**) Impact of a cell density on FQ efficacy. LNCaP cells were plated at the indicated densities 24 hours before a 24-hour treatment with FQ (20 μM). **(E**) FQ IC50 values (48 h treatment) in normal and cancer cell lines. (**F**) Clonogenic survival of LNCaP cells following treatment with vehicle, CQ (7 μM) or FQ (7 μM) for 24 h (n = 5); Mean ± SEM; paired t-test; **P < 0.01; ***P < 0.001. **(G**) Cell cycle analysis in LNCaP cells treated with vehicle, CQ (7 μM) or FQ (7 μM) for 72 h (n = 3).

Recently, in *The Lancet Infectious Diseases* Held J. *et al*. reported the results from phase 2 clinical study of a new third-generation antimalarial drug, ferroquine (FQ; SSR97193)^14,15^. FQ, a new analogue of CQ, showed promising efficacy and safety profile both as monotherapy and in combination with artesunate^14^. Interestingly, unlike all the other CQ analogues currently used for malaria treatment, FQ represents an organometallic compound in which ferrocene molecule (an iron atom sandwiched between two aromatic rings) is covalently bound to a 4-aminoquinoline and a basic alkylamine (Fig. 1A)^15–18^. FQ has strong antimalarial activity that greatly surpasses that of CQ, and it is active against CQ-resistant and multiresistant parasite strains^14^. However, up to date the anticancer properties of FQ remained unexplored.

Here, we assessed the potential application of FQ as an anticancer drug and investigated the mechanisms by which FQ targets cancer cells.

## Results

### Ferroquine induces proliferative arrest and cancer cell death

Analysis by light microscopy revealed significant morphological alterations as well as pronounced death of the lymph node carcinoma of the prostate (LNCaP) cells following treatment with FQ (15 μM, 24 h) (Fig. 1B). Further, analysis of cell viability by MTS assay demonstrated that FQ reduced viability of LNCaP cells in a dose- and time-dependent manner (Fig. 1C). This effect of FQ was much more pronounced compared to its parent compound chloroquine (CQ). Moreover, the reduction in cell viability induced by FQ was associated with significant cytolysis, as revealed by the release of the stable cytoplasmic enzyme, lactate dehydrogenase (LDH) into the cell-culture supernatant (Fig. 1C). It should be noted, however, that these effects of FQ depended on cell seeding density with a significant decrease of FQ efficacy on cells at high density (>15000 cells/cm^2^) (Fig. 1D). In addition to LNCaP cells, FQ effectively reduced the viability of all other cancer cell lines tested (prostate cancer cell lines PC3M, C4-2, PC3, DU-145; pancreatic cancer cell lines MiaPaCa2, Panc1; breast cancer cell line MCF7 (data not shown)) with IC50 values in a low micromolar range (Fig. 1E, Supplementary Table 1, Supplementary Figure S1A). FQ also reduced the viability of immortalized human normal prostate epithelial cells (PNT1A), although these cells tended to be more resistant to FQ treatments compared to most prostate cancer cell lines (Fig. 1E). Out of five prostate cancer cell lines tested, only PC3M cell line (highly metastatic androgen-independent prostate carcinoma cell line) appeared to be more resistant to FQ than normal PNT1A cells (Fig. 1E). We further confirmed cytotoxic properties of FQ by assessing its effect on cancer cell clonogenicity. Following treatment with CQ (7 μM) or FQ (7 μM) for 24 h, LNCaP cells were washed and left to recover for 10 days. FQ treatment resulted in almost complete loss of clonogenic survival. This effect of FQ greatly surpassed that of CQ (Fig. 1F). In fact, the decrease in cell number induced by FQ at low concentrations (<10 μM) was mainly caused by proliferation arrest rather than by cell death. Therefore, we next analyzed whether FQ affects cell cycle of LNCaP cells. Flow cytometry analysis revealed that FQ (7 μM, 72 h) reduced the proportion of LNCaP cells in S and G2/M phases of cell cycle, thus inducing proliferative blockade via cell cycle arrest in G0/G1-phase (Fig. 1G). To bolster the results obtained on LNCaP cells, we repeated all the experiments on less-sensitive PC3M cells (Supplementary Figure S1A, S1B, S1C). Of note, ferrocene (or aminoferrocene) alone or in combination with CQ (equimolar concentrations) did not induce PC3M prostate cancer cell death (Supplementary Figure S1D). We also tested the effect of ferrocene and ferrocene + CQ on the viability of PNT1A, C42, LNCaP and PC3 cells, and in all the cases treatments were without significant effect on cell viability (data not shown).

Overall, our results confirmed, that FQ as a monotherapy effectively provokes proliferative arrest and cancer cell death *in vitro*.

### Ferroquine induces caspase-independent apoptosis-like cell death and sensitizes cancer cells to stress

**Figure 2:**
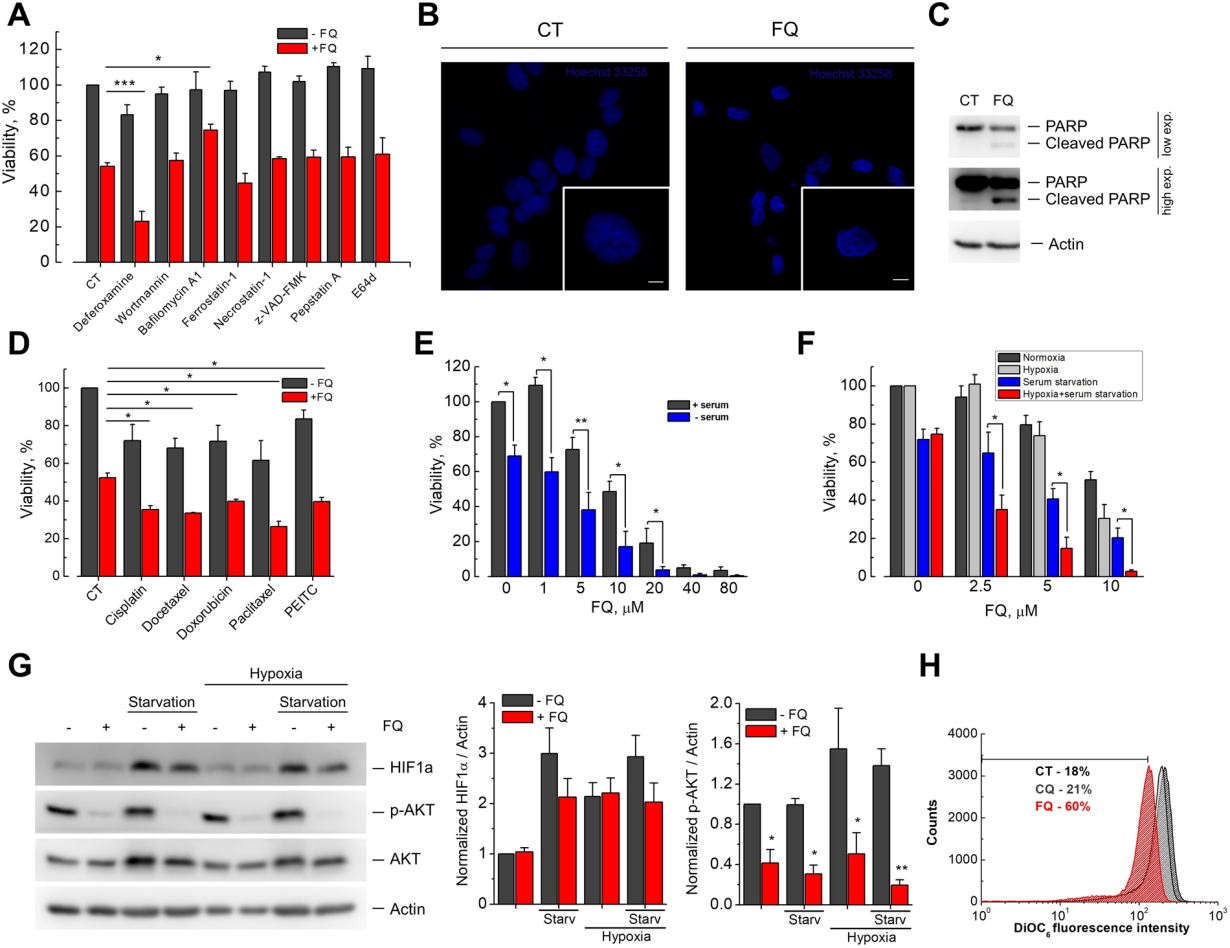
Ferroquine induces caspase-independent apoptosis-like cell death and sensitizes cancer cells to stress. (**A**) Viability (MTS) assay showing the effect of different cell-death inhibitors on LNCaP cells treated with FQ (10 μM) for 48 h (n = 3–6). Mean ± SEM; t-test; *P < 0.05; ***P < 0.001. (**B**) LNCaP cell nuclear morphology following treatment with vehicle or FQ (20 μM) for 24 h as revealed by Hoechst 33258 staining. Inset shows a representative cell. Scale bar, 5 μm. **(C)** Effect of FQ on PARP cleavage in LNCaP cells. (**D**) Viability (MTS) assay showing chemosensitization of LNCaP cells by FQ (n = 3). Mean ± SEM; t-test; *P < 0.05. (**E**) Effect of FQ on LNCaP cell viability in complete or serum-starved media (MTS, n = 4). Mean ± SEM; paired t-test; *P < 0.05; **P < 0.01. **(F**) Effect of FQ on LNCaP cell viability in normoxic and hypoxic conditions (MTS, n = 4). Mean ± SEM; t-test; *P < 0.05. (**G**) FQ negatively regulates Akt kinase and HIF-1α in LNCaP cells (n = 3). Mean ± SEM; t-test; *P < 0.05. (**H**) Flow cytometry experiment demonstrating increase in number of depolarized mitochondria induced by FQ (15 μM, 15 h) in LNCaP cells (n = 2).

Different types of cell death have been described, including necrosis, apoptosis, necroptosis and others^19^. Recently, the existence of an iron-dependent form of nonapoptotic cell death, called ferroptosis, has been demonstrated^20^. Considering that FQ contains iron in its structure we reasoned that FQ could induce cell death by ferroptosis. To test this hypothesis LNCaP cells were treated for 48 h with FQ (10 μM) alone or in combination with either ferroptosis specific inhibitor ferrostatin-1 (20 μM) or iron chelator deferoxamine (50 μM). However, neither ferrostatin-1 nor deferoxamine did not prevent FQ-induced cell death. Moreover, cotreatment with deferoxamine significantly potentiated cell death induced by FQ (Fig. 2A). Further, we tested the ability of different established cell death inhibitors to prevent FQ-induced cell death. LNCaP cells were pretreated with different cell death inhibitors for 3 h and then FQ (10 μM final) was added to the media for the next 45 h. Further, analysis of cell viability by MTS assay demonstrated that FQ-induced cell death was not significantly affected by inhibitors of caspases (z-VAD-FMK, 20 μM), RIPK1 (necrostatin-1, 30 μM), cathepsins (Pepstatin A and E64d, 10 μM), and autophagy (wortmannin, 100 nM), suggesting that FQ-induced cell death type is distinct from ferroptosis, caspase-dependent apoptosis, necroptosis, or autophagy (Fig. 2A). In contrast, bafilomycin A1 (50 nM), an inhibitor of vacuolar-type H^+^-ATPase (V-ATPase), reduced cell death induced by FQ, suggesting the possible role for lysosomes in FQ-induced cell death (Fig. 2A).

Although caspase inhibitor z-VAD-FMK did not prevent FQ-induced cell death, morphological characteristics of FQ-treated cells resembled hallmarks of apoptosis-like programmed cell death. These include plasma membrane blebbing, cell shrinkage, rounding and detachment following treatment with FQ. Moreover, assessment of LNCaP cell nuclear morphology using Hoechst 33258 nuclear dye revealed the increased number of condensed and moderately condensed convoluted nuclei (another hallmark of apoptosis) following treatment with FQ (Fig. 2B and Supplementary Figure S2A). Finally, treatment of LNCaP cells with FQ (25 μM) for 24 h resulted in appearance of a cleaved poly (ADP-ribose) polymerase-1 (PARP-1) 89-kD fragment characteristic of apoptosis (Fig. 2C). Overall, these results suggest that FQ induces caspase-independent apoptosis-like cancer cell death which depends on the activity of V-ATPase.

Considering pronounced cancer cell-killing effect of FQ as a single agent *in vitro*, we next assessed whether FQ modulates cancer cell vulnerability to stress, such as chemotherapy treatments, serum starvation or hypoxia. Indeed, CQ has been previously reported to enhance cancer cell killing by chemotherapy and radiation therapy. To determine whether FQ also has a potential to be used as an adjuvant to anticancer chemotherapy, we treated LNCaP cells for 48 h with different chemotherapeutic drugs (cisplatin 5 μM, docetaxel 2.5 nM, doxorubicin 25 nM, paclitaxel 5 nM and phenethyl isothiocyanate (PEITC) 10 μM) alone or in combination with FQ (10 μM). For combination treatments LNCaP cells were first incubated with chemotherapeutic drugs for 12 h and then FQ was added to the medium for the last 36 h. Analysis of cell viability by MTS assay revealed modest but statistically significant reduction in cell viability in the case of combined treatments with FQ and chemotherapy drugs compared to chemotherapy or FQ treatments alone (Fig. 2D). These results suggest that FQ could be considered as an adjuvant to existing anticancer chemotherapy.

To test whether FQ sensitizes cancer cells to serum starvation we treated LNCaP cells for 48 h with FQ (different concentrations) in the presence or absence of serum in culture media. The effect of FQ was significantly potentiated in serum-free conditions (Fig. 2E). Moreover, the number of condensed nuclei significantly increased when LNCaP cells were treated with FQ in the absence of serum in culture media (data not shown). In contrast, CQ did not show any significant changes in its cytotoxicity in serum-free conditions (Supplementary Figure S2B).

It is well established that solid tumors typically develop harsh microenvironments characterized by the presence of regions with poor nutrient and oxygen supply. The cells in these hypoxic regions acquire an aggressive phenotype and become resistant to both radiotherapy and chemotherapy^21^. Therefore, we next investigated whether FQ will be able to induce cancer cell death in hypoxic conditions. LNCaP cells were incubated in normoxic (20% O_2_) or hypoxic (1.2% O_2_) conditions for 48 h in the absence or presence of serum in culture media. During this incubation time hypoxia did not induce any significant changes in cell viability neither in control nor in serum-starved conditions compared to normoxic control and serum-starved conditions, respectively (Fig. 2F). In hypoxic nutrient-rich conditions FQ (tested at different concentrations) tended to be more effective, compared to normoxic conditions, however the difference did not reach statistical significance (Fig. 2F). In contrast, LNCaP cells incubated in serum-starved medium in hypoxia showed greatly increased sensitivity to FQ compared to cells incubated in normoxic serum-starved conditions (Fig. 2F). The same effect was observed in other cell lines tested (C4-2 and PC3M, data not shown). This phenomenon was absent when cells were treated with CQ instead of FQ, suggesting that some specific properties of FQ are important here (Supplementary Figure S2C).

Considering that Akt phosphorylation is widely established to be one of the key events involved in the regulation of prostate cancer cell survival^22^, we next tested whether FQ influence Akt activation in prostate cancer cells. Treatment of LNCaP cells with FQ (30 μM) for 2 h in the presence or absence of serum in culture media resulted in a striking decrease in phosphorylated Akt levels, suggesting that FQ acts as Akt inhibitor in prostate cancer cells (Fig. 2G). Further, the results of our experiments in starved and hypoxic conditions encouraged us to test whether FQ could interfere with expression/stabilization of hypoxia-inducible factor-1α (HIF-1α) in prostate cancer cells. In fact, HIF-1α has been previously reported to be a key survival factor for serum- or oxygen-deprived prostate cancer cells^23,24^. Serum-starvation for 2 h in normoxic or hypoxic (1.2% O_2_) conditions induced HIF-1α accumulation in LNCaP cells. Treatment of LNCaP cells with FQ (30 μM) for 2 h reduced HIF-1α expression, suggesting that FQ has HIF-1α inhibitory activity in serum-starved conditions (Fig. 2G). Of note, FQ was ineffective in reducing HIF-1α levels induced by hypoxia in nutrient-reach conditions (Fig. 2G).

Previous studies reported that aminoquinolines could affect mitochondrial function, and in this way sensitize cancer cells to metabolic stress^11,25^. We therefore next tested whether FQ caused alterations in mitochondrial transmembrane potential (ΔΨ) in prostate cancer cells by using ΔΨ-sensitive probe 3,3′-Dihexyloxacarbocyanine Iodide (DiOC6(3)). We treated LNCaP and PC3M cells with vehicle, CQ (15 μM) or FQ (15 μM) for 15 h, stained with DiOC6(3) dye and analyzed its fluorescence intensity by flow cytometry. This analysis revealed that FQ induced a moderate yet significant decrease in ΔΨ (Fig. 2H, Supplementary Figure S2D). We confirmed this result by using another ΔΨ-sensitive dye tetramethyl-rhodamine ethyl ester (TMRE) (Supplementary Figure S2E). This data suggests that FQ compromises mitochondrial function in prostate cancer cells and in this way can potentially sensitize cancer cells to metabolic stress.

### Ferroquine arrests autophagy

**Figure 3:**
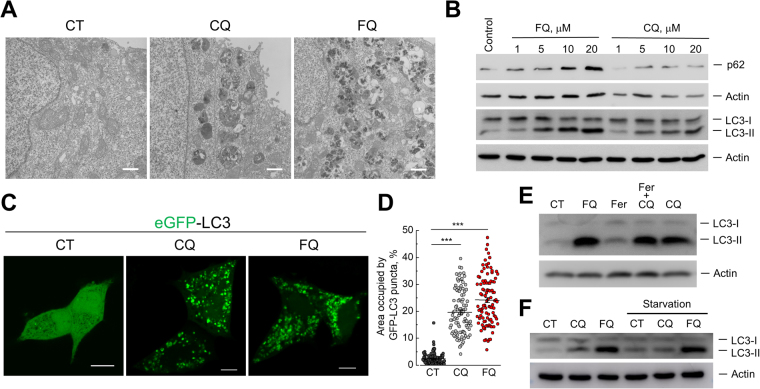
Ferroquine arrests autophagy. (**A**) Transmission electron microscopy images of LNCaP cells treated with vehicle, CQ (10 μM) or FQ (10 μM) for 24 h. Scale bar, 1 μm. **(B)** Immunoblotting for LC3, p62 and Actin as a loading control. LNCaP cells were treated with CQ or FQ for 12 h. (**C)** Representative confocal images of LNCaP cells transfected with eGFP-LC3 and treated as in (**A**). Scale bars, 10 μm. **(D)** Quantification of (**C**) (n = 100). Mean ± SEM; Mann-Whitney test; ***P < 0.001. **(E)** Effect of FQ (20 μM), ferrocene (20 μM) and ferrocene + CQ (20 μM) on autophagy in LNCaP cells. Immunoblotting for LC3 and Actin. **(F)** Immunoblotting for LC3 and Actin. LNCaP cells were treated with vehicle, CQ (10 μM) or FQ (10 μM) for 6 h in complete medium or in glucose-free salt solution. Full-length blots are included in the Supplementary Information.

FQ induced obvious changes in cellular morphology within hours of treatment. Live-cell imaging by light microscopy revealed the accumulation of cytoplasmic vacuoles, cell rounding and detachment of FQ-treated LNCaP cells. TEM analysis confirmed significant accumulation of expanded single-membraned vacuoles (containing multi-lamellar membrane structures, lucent vesicles and electron dense material) and their aggregates following treatment with FQ (Fig. 3A, Supplementary Figure S3A). This effect greatly resembled previously reported effects of lysosomotropic compounds (e.g. CQ) blocking autophagy at late stages and inducing accumulation of autophagic vacuoles^26,27^. Of note, no accumulation of free autophagosomes (double-membraned vesicles) has been observed in response to treatment with FQ (Fig. 3A, Supplementary Figure S3A).

To test whether FQ induces accumulation of autophagic markers in LNCaP cells we analyzed the levels of endogenous LC3-II protein by western blot (LC3 is the most widely used marker of autophagy^28^). LNCaP cells were treated with different concentrations of FQ or CQ for 12 h. LC3 immunoblotting analysis demonstrated that FQ induced accumulation of LC3-II protein in LNCaP cells in a dose-dependent manner (Fig. 3B). This accumulation was more pronounced than that induced by CQ. Immunoblot analysis of another autophagic marker, p62 (which is autophagy substrate protein degraded by autophagy), showed its accumulation in a dose-dependent manner following CQ or FQ treatments indicating that autophagy is inhibited by FQ and autophagy substrates are not degraded (Fig. 3B). Of note, FQ also caused an increase in LC3-II levels in prostate cancer PC3 and PC3M cells but not in DU-145 cells lacking full-length autophagy-related gene 5 (ATG5) (which is essential for LC3 lipidation), suggesting the necessity of the functional autophagic lipidation machinery (Supplementary Figure S3B).

Next to confirm accumulation of autophagic markers in LNCaP cells the cells were transfected with eGFP-LC3, treated with vehicle, CQ (10 μM) or FQ (10 μM) for 24 h and eGFP fluorescence was analyzed by confocal microscopy. Vehicle-treated LNCaP cells exhibited diffused cytosolic eGFP fluorescence with occasional puncta (representing autophagosomes) (Fig. 3C). Both CQ and FQ treatments resulted in accumulation of puncta and decrease in diffused fluorescence intensity, suggesting accumulation of autophagic vacuoles (Fig. 3C). Moreover, FQ treatment resulted in accumulation of aggregates of puncta, thus making their counting rather difficult. Therefore we quantified these results by assessing the area occupied by GFP-LC3 puncta per cell (Fig. 3D). Interestingly, variation of fluorescence intensity (spots with high fluorescence intensity within big puncta with lower fluorescence intensity) was observable within individual large puncta following treatments with CQ or FQ (Fig. 3C, Supplementary Figure S3C).

It should be noted, that ferrocene induced subtle changes in autophagy in LNCaP cells (Fig. 3E). However, in combination with CQ ferrocene induced accumulation of LC3-II levels comparable to that induced by FQ (Fig. 3E).

We further tested whether FQ can block starvation-induced autophagy. LNCaP cells were treated with glucose-free HEPES-buffered salt solution (HBS) with or without FQ (10 μM) or CQ (10 μM) for 12 h and LC3-II levels were analyzed by immunoblotting (Fig. 3F). HBS-treated cells showed increased levels of LC3-II protein, suggesting activation of autophagy by HBS. FQ cotreatment further increased LC3-II levels induced by HBS, whereas CQ failed to do this (probably because of low concentration, which is not enough to completely block lysosomal function) (Fig. 3F). These results indicate, that FQ arrests both basal and induced autophagy.

### Ferroquine blocks autophagic flux

**Figure 4:**
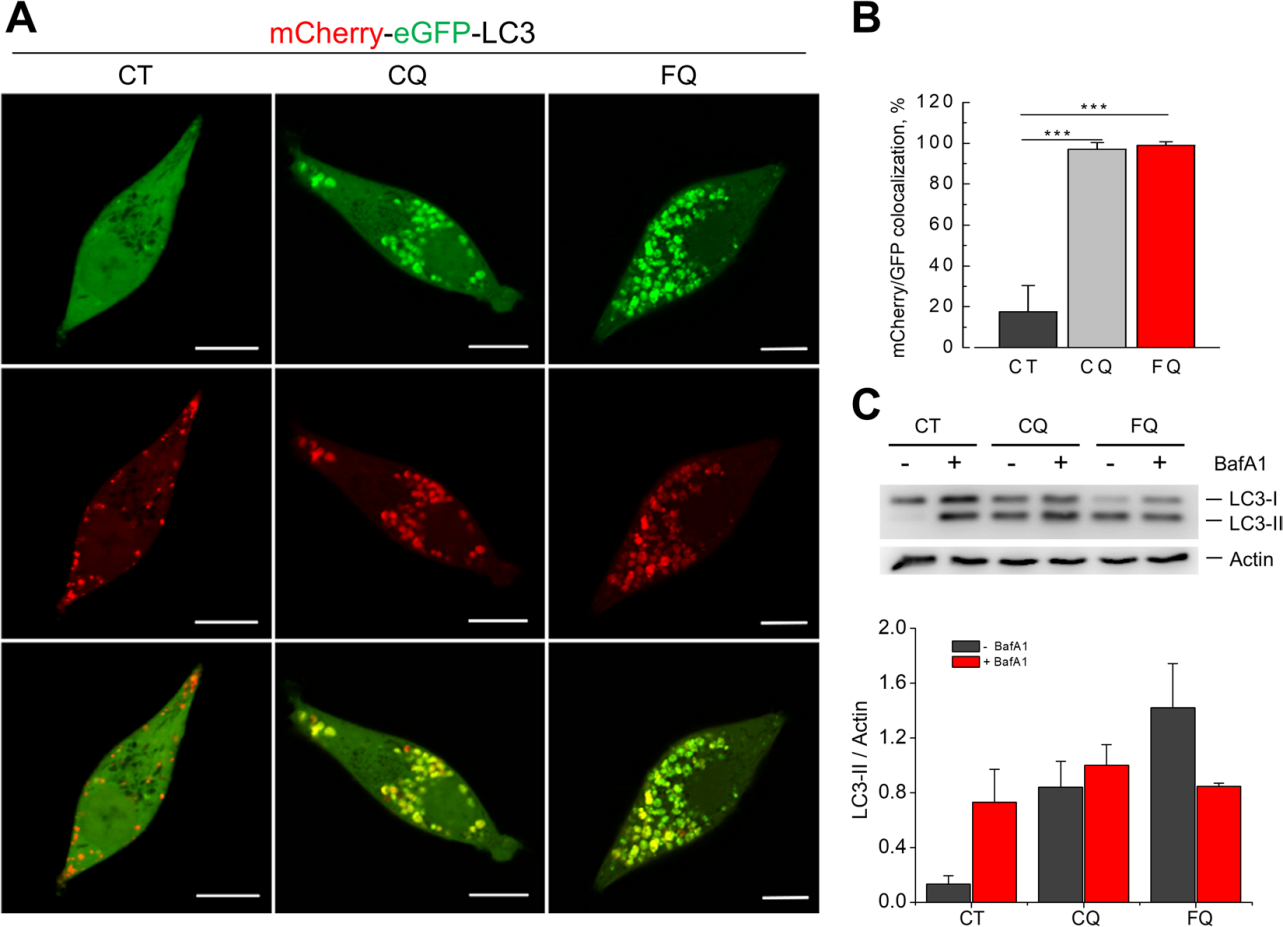
Ferroquine blocks autophagic flux. (**A**) Autophagic flux analysis using tandem mCherry-eGFP reporter assay. LNCaP cells transfected with mCherry-GFP-LC3B were treated with vehicle, CQ (5 μM) or FQ (5 μM) for 24 h. Representative confocal images are shown. Scale bar, 10 μm. **(B)** Quantification of (**A**) (n = 22). Mean ± SD; t-test; ***P < 0.001. **(C)** Autophagic flux analysis using lysosomal inhibitor bafilomycin A1. LNCaP cells were pretreated with bafilomycin A1 (100 nM, 1 h) and then co-treated with vehicle, CQ (20 μM) or FQ (20 μM) for 3 h. (n = 3). Mean ± SEM.

To confirm inhibitory action of FQ on autophagy we analyzed autophagic flux in LNCaP cells using a tandem mCherry-eGFP reporter fluorescence assay. LNCaP cells were transfected with mCherry-eGFP-LC3B plasmid and in 48 h treated with vehicle, CQ (5 μM) or FQ (5 μM) for 24 h. The colocalization of GFP and mCherry signals was analyzed by confocal microscopy. In control conditions most of the puncta were red-only, confirming that autophagosomes are degraded by acidic lysosomes, and therefore the eGFP signal is quenched (Fig. 4A,B). In contrast, CQ and FQ induced formation of both eGFP- and mCherry-positive puncta, indicating that CQ and FQ induce accumulation of autophagic vacuoles with impaired acidification, thus eGFP signal is not quenched anymore (Fig. 4A,B).

Next, we confirmed inhibition of autophagic flux by FQ by using lysosomal inhibitor bafilomycin A1. Bafilomycin A1 (100 nM, 1 h) alone effectively induced accumulation of LC3-II in LNCaP cells (Fig. 4C). However, pretreatment with bafilomycin A1 failed to potentiate FQ-induced LC3-II level, suggesting that FQ inhibits LC3-II degradation at the autolysosomal level and activity of lysosomal V-ATPase can regulate this process (Fig. 4C).

### Ferroquine provokes lysosomal enlargement and distortion

**Figure 5:**
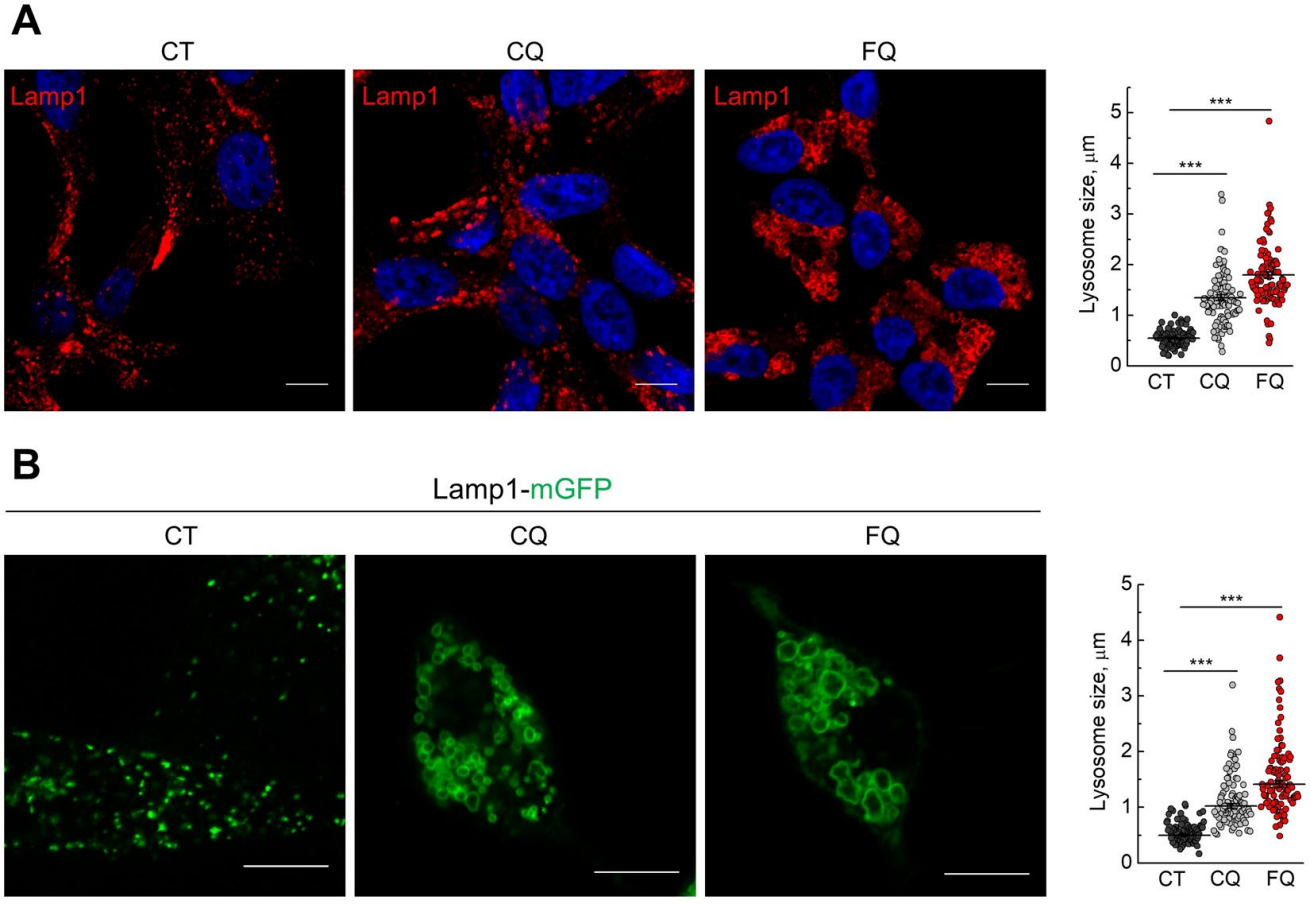
Ferroquine provokes lysosomal enlargement and distortion. (**A**) LNCaP cells were treated with vehicle, CQ (5 μM) or FQ (5 μM) for 12 h and immunostained for Lamp1. Representative confocal images and lysosome size quantification are shown (n = 100); Scale bar, 10 μm. Mean ± SEM; t-test; ***P < 0.001. (**B**) LNCaP cells transfected with Lamp1-mGFP were treated with vehicle, CQ (5 μM) or FQ (5 μM) for 12 h and analyzed by confocal microscopy. Representative confocal images and lysosome size quantification are shown. (n = 100); Scale bar, 10 μm. Mean ± SEM; t-test; ***P < 0.001.

Accumulation of mCherry-GFP-LC3B-positive puncta following treatment with FQ indicates that FQ could impair lysosomal function. To test this, we first assessed lysosomal morphology in control and FQ- or CQ-treated LNCaP cells. LNCaP cells were treated with CQ (5 μM) or FQ (5 μM) for 12h and lysosomal-associated membrane protein 1 (Lamp1, lysosomal marker) immunofluorescence was then analyzed by confocal microscopy (Fig. 5A). Alternatively, LNCaP cells were transfected with Lamp1-mGFP and in 48 h were treated and analyzed as mentioned above (Fig. 5B). Both CQ- and FQ-treated cells contained remarkably larger lysosomes with FQ-treated lysosomes being the largest. Moreover, following treatment with FQ many lysosomes exhibited distorted shape (Fig. 5A,B).

### Ferroquine alters lysosomal pH and induces lysosomal membrane permeabilization

**Figure 6:**
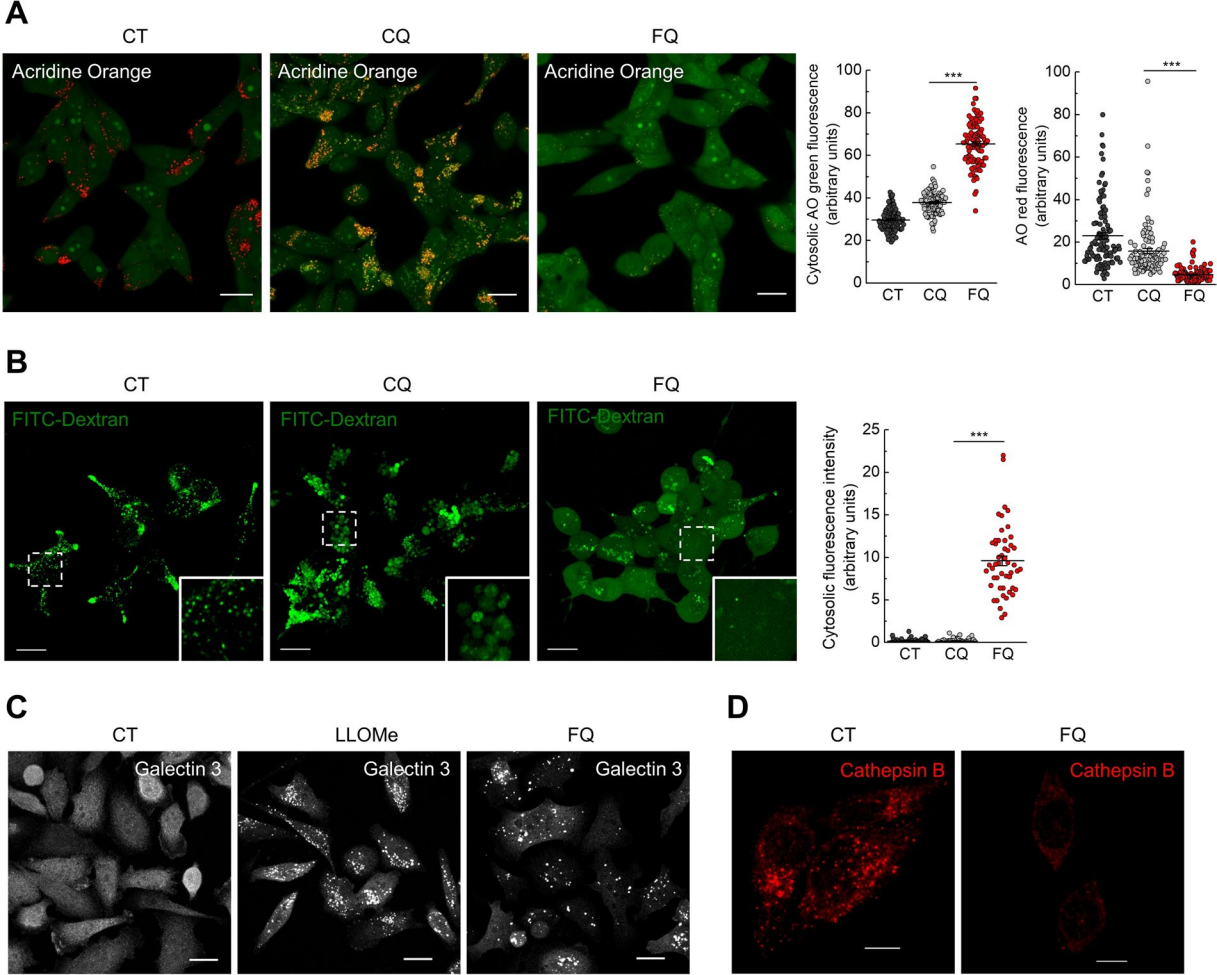
Ferroquine alters lysosomal pH and induces lysosomal membrane permeabilization. (**A**) FQ decreases lysosomal acidification and induces relocation of acridine orange from acidic vesicles to cytosol. LNCaP cells were stained with acridine orange, washed and treated with vehicle, CQ (30 μM) or FQ (30 μM) for 1 h. Representative confocal images and quantification of cytosolic green and red fluorescence are shown (n = 100 cells). Scale bar, 20 μm. Mean ± SEM; Mann-Whitney test; ***P < 0.001. **(B)** FQ induces release of FITC-dextran from lysosomes. LNCaP cells were treated with vehicle, CQ (20 μM) or FQ (20 μM) for 12 h. Representative confocal images and quantification of cytosolic fluorescence intensity are shown (n = 50 cells). Scale bar, 20 μm. Mean ± SEM; Mann-Whitney test; ***P < 0.001. **(C)** FQ induces translocation of cytosolic galectin 3 to damaged lyzosomes. PC3 cells were treated with vehicle, 2 mM LLOMe or FQ (25 μM) for 2 h. Representative confocal images are shown. **(D)** FQ induces cathepsin B release. PC3 cells were treated with FQ (25 μM) for 2 h. Representative confocal images are shown.

To test whether FQ affects lysosomal acidification, LNCaP cells were stained with acridine orange (an acidotropic dye that is widely used as an indicator of endolysosomal acidification), washed and treated with vehicle, FQ (30 μM) or CQ (30 μM) for 1 h. Acridine orange fluorescence was analyzed by confocal microscopy. Non-treated LNCaP cells exhibited cytosolic green fluorescence (representing neutral environment) with red puncta (representing acidic vesicles). Treatment with CQ resulted in accumulation of enlarged puncta emitting in both green and red, indicating deacidification of vesicles (Fig. 6A). Treatment with FQ lead to a significant decrease in red fluorescence with concomitant increase in cytosolic green fluorescence, indicating increase in vesicular pH and relocation of acridine orange from vesicles to cytoplasm (Fig. 6A). This result led us to a hypothesis that FQ could induce lysosomal membrane permeabilization (LMP) in LNCaP cells. To test this hypothesis we assessed FQ-induced LMP by fluorescent dextran release assay^29^. LNCaP cells were incubated with FITC-dextran (10 kDa, 1 mg/ml) for 7 h, washed and chased for 3 h in complete medium. Then cells were treated with vehicle, FQ (20 μM) or CQ (20 μM) for 12 h and analyzed by confocal microscopy. In control non-treated cells FITC-dextran appeared in puncta, consistent with its reported localization in lysosomes (Fig. 6B). CQ treatment significantly increased the size of FITC-dextran-positive puncta. In contrast, FQ treatment resulted in diffused staining pattern throughout the cytosol with occasional puncta, indicating release of FITC-dextran from lysosomes into the cytosol (Fig. 6B). These results demonstrate that FQ but not CQ induces LMP in LNCaP cells. Next, we confirmed the LMP inducing activity of FQ by lysosomal galectin puncta assay^29,30^. Galectins are sugar-binding proteins, which translocate from the cytosol to lysosomes upon LMP. We performed this assay on PC3 cells by using galectin-3 antibodies (LNCaP cells, which were used throughout this study, do not express galectin-3). PC3 cells were treated with vehicle, 2 mM L-leucyl-L-leucine-methyl ester (LLOMe, a well-known LMP inducer) or FQ (25 μM) for 2 h, and galectin-3 immunofluorescence was then analyzed by confocal microscopy (Fig. 6C). Control cells exhibited diffused cytosolic fluorescence, whereas both LLOMe and FQ treatments resulted in punctate galectin-3 staining, suggesting galectin-3 translocation to damaged lysosomes (Fig. 6C). We further assessed cathepsin B localization, as another marker of LMP. PC3 cells were treated with FQ (25 μM) for 2 h, and cathepsin B immunofluorescence was then analyzed by confocal microscopy (Fig. 6D). In contrast to control untreated cells displaying punctate cathepsin B staining, FQ-treated cells exhibited diffused cathepsin B staining (with lower intensity) with almost no puncta present in the cells (Fig. 6D). Overall, these results show that FQ provokes lysosomal distortion, alters lysosomal pH and induces lysosomal membrane permeabilization in prostate cancer cells.

### Ferroquine inhibits LNCaP-derived xenograft growth *in vivo*

**Figure 7:**
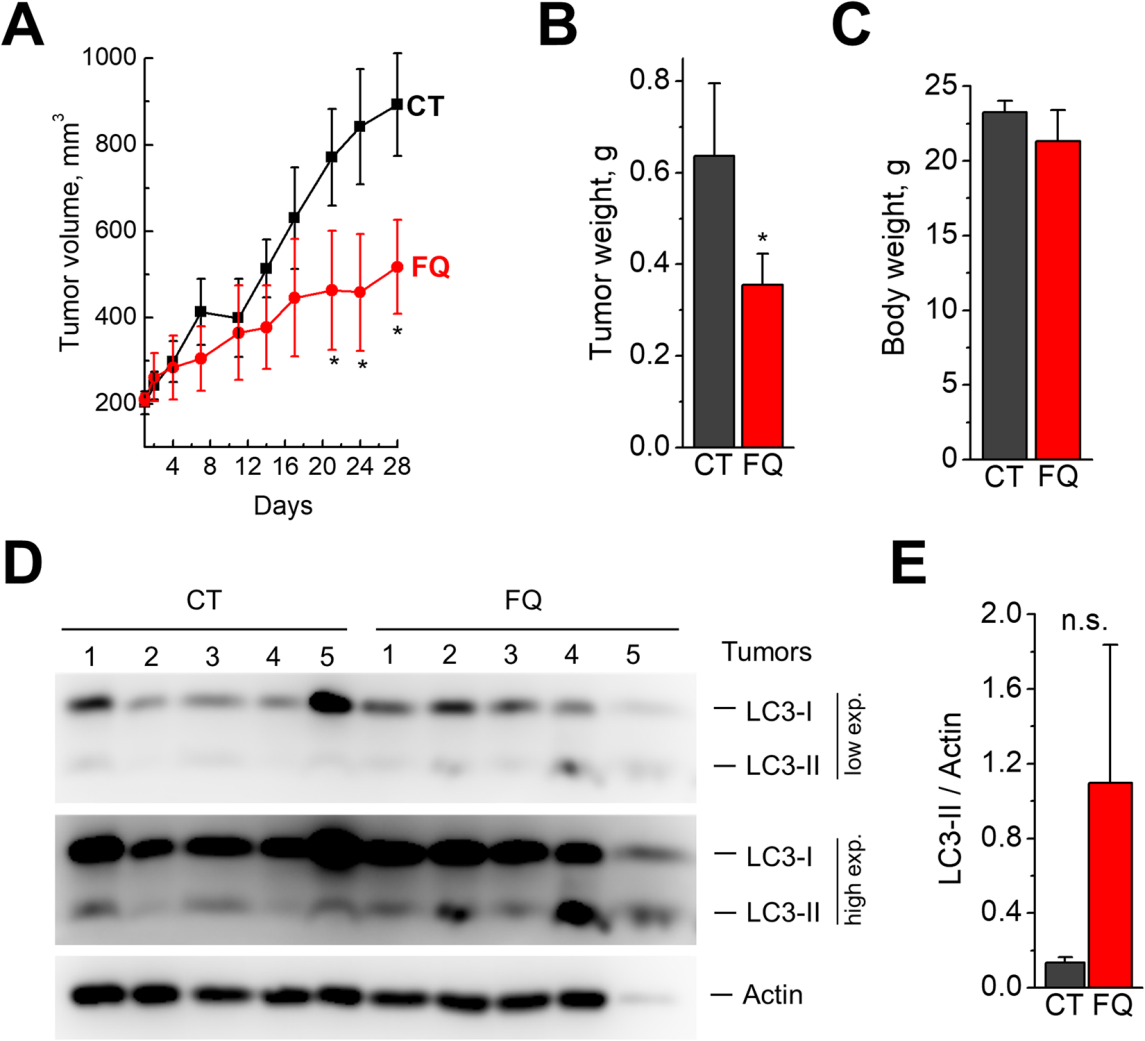
Ferroquine inhibits LNCaP-derived xenograft growth *in vivo*. (**A)** *In vivo* antitumor effect of FQ (n = 5). Data presented as Mean ± SD. Mann-Whitney test; *P < 0.05. **(B)** Effect of FQ on tumor weight. Data presented as Mean ± SD. Mann-Whitney test; *P < 0.05. (**C**) Effect of FQ on mice body weight. Data presented as Mean ± SEM. (**D**) Immunoblotting for LC3 and Actin in lysates from individual vehicle-treated or FQ-treated tumors. **(E**) Quantification of LC3II/Actin ratio. Data presented as Mean ± SEM.

Considering the pronounced effect of FQ on cancer cell proliferation and viability *in vitro*, we next assessed whether FQ, as a single agent, could impair tumor growth *in vivo*. To investigate antitumorigenic potential of FQ *in vivo*, we selected two prostate cancer cell lines: LNCaP, an androgen-dependent cell line representing early-stage prostate cancer; and PC3M, highly metastatic androgen-independent cell line representing late-stage prostate cancer. Of note, LNCaP cells were the most sensitive and PC3M cells the least sensitive to FQ out of all prostate cancer cell lines tested *in vitro*. Treatments with FQ (50 mg/kg) or vehicle alone (see Materials and Methods) was started when tumor volume reached 150–200 mm^3^ (for PC3M – in 11 days after tumor inoculation; for LNCaP – in 24 days after tumor inoculation). Mice were treated daily for 5 days with 2 days off treatment for the total period of 22 days, in the case of PC3M-derived tumors, or 28 days in the case of LNCaP-derived tumors. LNCaP-tumor growth was significantly impaired in FQ-treated mice compared to vehicle-treated animals (Fig. 7A,B). The treatment was well-tolerated with non-significant reduction in animal body weight and no premature death (Fig. 7C and Supplementary Figure S4C). Immunoblot analysis of LC3 protein expression revealed higher LC3-II/Actin levels in FQ treated LNCaP-derived tumors compared with vehicle-treated tumors, however the difference have not reached statistical significance (Fig. 7D,E). Nevertheless, this result shows that FQ has a clear tendency to inhibit autophagy *in vivo*.

In contrast to LNCaP-derived tumors, the growth of PC3M-derived tumors was not affected by FQ (Supplementary Figure S4A, B). PC3M-derived tumors were growing much faster than LNCaP-derived tumors, so we stopped the experiment much earlier to prevent excessive tumor growth and animal suffering.

Overall, these results suggest that FQ has antitumorigenic potential *in vivo*, however the final effect of FQ on tumor growth *in vivo* could apparently depend on tumor type and stage.

## Discussion

Drug repositioning (new uses for old drugs) has recently gained considerable attention of scientists as promising strategy for accelerated development of new anticancer therapies^31^. Thus, numerous drugs originally designed as antidiabetic, analgesic, antihypertensive, antibiotic, antiepileptic and antimalarial have been tested for their anticancer activity^32^. Out of these drugs, the development and repurposing of antimalarials is now considered as a promising direction for the elaboration of effective anticancer therapies.

Here we present ferroquine (FQ), the next generation antimalarial drug, as a promising candidate for repositioning as cancer therapeutics. FQ, a new analogue of chloroquine (CQ), represents an organometallic compound in which ferrocene molecule (an iron atom sandwiched between two aromatic rings) is covalently bound to a 4-aminoquinoline and a basic alkylamine (Fig. 1A)^15–18^. The encouraging results from phase 2 clinical study show that FQ is safe and effective against CQ-resistant and multiresistant parasite strains both as monotherapy and in combination with artesunate^14^. FQ is the only candidate in phase 2 development that has a half-life and minimum inhibitory concentration that lasts more than 20 days and it is not affected by food^14,33^. FQ has been shown to be generally well-tolerated up to 1600 mg as single dose and up to 800 mg as repeated dose^33,34^. All these results indicate that FQ has tremendous potential to be utilized in clinics.

It should be noted, however, that ferrocene (the core of FQ) itself is not particularly toxic with oral LD50 value of 832 mg/kg for mice. In line with previous studies, we did not observe any significant effect of ferrocene alone or in combination with CQ on prostate cancer cell viability^35^. In contrast, multiple ferrocene-containing molecules has been previously reported to have anticancer activity and application of ferrocene derivatives in cancer therapy is an active area of research^36,37^. Noteworthy, organometallic compounds (e.g. cisplatin, carboplatin, oxaliplatin) are well known for their anticancer activity and are now in clinical use^38,39^. Thus, organometallic nature, presence of ferrocene core and strong antimalarial activity that greatly surpasses that of CQ suggest that FQ could potentially possess significant anticancer activity.

Our results demonstrate that FQ effectively reduced the viability of different cancer cell types (prostate, pancreatic and breast) with IC50 values in a low micromolar range.

We demonstrate that effective cancer cell death induced by FQ involves several factors including negative regulation of Akt kinase and HIF-1α, mitochondrial impairments, inhibition of autophagic-lysosomal function and LMP. Nevertheless, further work is necessary to understand the mechanisms by which FQ exerts its lysosomal and extralysosomal functions. FQ effectively induced cancer cell death independent of their p53 status and hormonal-dependence. Androgen-dependent LNCaP cells harboring wild-type p53 as well as androgen-independent PC3 and DU-145 cells harboring non-functional p53 – in all these cell lines FQ effectively induced cell death. Of note, FQ also reduced the viability of normal prostate epithelial cells PNT1A with IC50 = 22 μM. Although this IC50 value is greater compared to that of most prostate cancer cell lines tested, we cannot conclude that FQ exhibits strong cancer cell selectivity *in vitro*. In fact, all the viability experiments on normal and cancer cell lines were conducted in identical nutrient reach normoxic conditions. In reality, many solid tumors (including prostate tumors) are characterized by harsh microenvironment with hypoxia and lack of nutrients^24^. Interestingly, we demonstrate that FQ is particularly effective in starved and hypoxic conditions which are frequently observed in solid cancers but are unusual and unnatural for normal tissues. Thus, at nontoxic concentration (for normal PNT1A cells under nutrient reach normoxic conditions) of 2.5 μM FQ induces about 60% LNCaP cell death in serum starved and hypoxic conditions. Therefore, we suppose that *in vivo* FQ primarily “selects” for starved and hypoxic cells. Apparently, negative regulation of prosurvival Akt kinase as well as HIF-1α by FQ plays an important role in FQ-induced prostate cancer cell death in serum starved and hypoxic conditions, as both Akt and HIF-1α have been previously reported to be key survival factors for serum- or oxygen-deprived prostate cancer cells^22–24^.

Importantly, we confirmed anticancer efficacy of FQ by performing *in vivo* experiments, in which FQ effectively inhibited LNCaP-derived xenograft growth in mice, establishing therapeutic potential of this molecule in cancer. It should be noted, however, that FQ failed to inhibit PC3M-derived xenograft growth. The reasons which stand behind these differences are not clear. Although LNCaP and PC3M cells are not at all comparable in terms of aggressiveness and metastatic potential and do not represent the same cancer stage (LNCaP cells represent early-stage androgen-sensitive prostate cancer, while PC3M cells represent highly metastatic late-stage androgen-independent prostate cancer), we cannot conclude that FQ can be regarded as a potential treatment specifically for early-stage prostate cancer. Considering that drug sensitivity is determined by multiple intrinsic genetic and epigenetic factors we cannot exclude the possibility that many metastatic/aggressive cancer cell lines could be more sensitive to FQ both *in vitro* and *in vivo* compared to PC3M cell line. For example, two androgen-independent prostate cancer cell lines (PC3 and DU-145) showed greater sensitivity to FQ *in vitro* in comparison with PC3M cell line. Thus, further studies are needed to reveal determinants of sensitivity to FQ.

Out of numerous antimalarial drugs exhibiting anticancer activity, just a few molecules have been reported to have strong single-agent antitumor activity *in vivo*. One of the most promising is Lys05, a novel dimeric derivative of chloroquine, which showed significant *in vivo* anticancer activity^12^. Most of other molecules, including CQ and HCQ, have been proposed as drugs sensitizing cancer cells to existing chemotherapy. However, significant toxicity at high doses and limited efficacy slows down their clinical application. An advantage of FQ is that its antimalarial and anticancer activity greatly surpasses that of CQ while clinical studies confirmed its safety. Our data suggest that FQ could also be considered as an adjuvant to anticancer chemotherapy, as FQ enhances the anticancer activity of several chemotherapeutics.

Cell death induced by FQ has been reduced by preincubation with an inhibitor of V-ATPase, bafilomycin A1, suggesting an important role for V-ATPase in this process. V-ATPase is localized on lysosomal membranes and has been reported to be the driving force of cationic drug uptake by cells, and inhibition of V-ATPase prevented lysosomal accumulation and cytotoxicity of numerous lysosomotropic drugs^40^. FQ, being a lipophilic weak base (pKa_1_ = 7; pKa_2_ = 8.45) also tends to accumulate in cellular acidic compartments^41^. By analogy with the suggested antimalarial mechanism of action we proposed that at neutral pH FQ in its unprotonated form enters the cell by passive diffusion due to its lipophilic properties. Then FQ enters acidic vacuoles (e.g. lysosomes), becomes protonated and trapped in these vacuoles^42,43^. This leads to vacuole deacidification and accumulation of undigested material. Indeed, our data clearly demonstrate the inhibition of lysosomal function by FQ. FQ induces lysosomal enlargement and shape distortion, deacidification as well as lysosomal membrane permeabilization (LMP), and cathepsin B release. Our results emphasize that FQ-induced lysosomal dysfunctions are more severe than that induced by CQ. The weaker base properties of FQ compared to CQ together with its higher lipophilicity at neutral pH as well as specific structure account for better potency for FQ to cross membranes and higher accumulation in acidic vacuoles^43^. FQ has been reported to be 100 times more lipophilic than CQ at neutral pH, and is expected to be accumulated in acidic vacuoles 50 times more efficiently than CQ^41^.

Naturally, inhibition of lysosomal function by FQ resulted in inhibition of autophagic flux. We show that FQ induces accumulation of large eGFP-LC3-positive vacuoles containing smaller puncta with high fluorescence intensity, presumably resulting from a series of fusion events between autophagosomes and nonfunctional lysosomes. These results correlate well with TEM images of FQ-treated cells, where multiple small vesicles are present in large vacuoles. It should be noted, however, that FQ effectively decreased viability of autophagy-defective DU-145 cells (lacking full-length ATG5), suggesting that at least in some cell types macroautophagy is not related to FQ-induced cell death. This hypothesis is consistent with the recent studies demonstrating autophagy-independent effects of CQ on cancer cell viability^44,45^.

Although our study revealed different intracellular targets of FQ, the specific molecular mechanisms underlying FQ-induced cancer cell death remain to be elucidated. Apparently, the lysosomes constitute the primary targets responsible for the effects of FQ on cell viability, as bafilomycin A1 reduced FQ-induced cell death. However, other factors, such as inhibition of Akt kinase should also be considered. Indeed, a dual inhibition of Akt kinase and autophagy appears to be an effective approach in cancer treatment^46^. Thus, a dual inhibition of Akt kinase and autophagic-lysosomal function by FQ could potentially be important for the final effects observed.

Overall, our results identified FQ as a promising new drug with a potent anticancer activity and our future studies will be focused on the elaboration of effective combination regimens involving FQ and novel chemotherapeutics.

## Materials and Methods

### Antibodies and reagents

Rabbit anti-LC3B (L7543), mouse anti-βActin (A5441), N-Acetyl-L-cysteine (A9165) were from Sigma. Mouse anti-p62 (D-3, sc-28359), mouse anti-Lamp1 (H4A3, sc-20011), mouse anti-HIF-1α (28b, sc-13515), rabbit anti-Cathepsin B (sc-6493), mouse anti-Galectin 3 (sc-32790), DL-α-Tocopherol (sc-294383A), H-Leu-Leu-OMe Hydrochloride (sc-285992A), Trolox (sc-200810), Ferrocene (sc-353607), Deferoxamine mesylate (sc-203331) were from Santa Cruz Biotechnology. Rabbit anti-PARP (9542S) were from Cell Signaling. Acridine orange (A-3568) was from Life Technologies. Bafilomycin A1 (1334) was from Tocris. Chloroquine (C6628), Wortmannin (W1628), Ferrostatin-1 (SML0583), Necrostatin-1 (N9037), Cisplatin (P4394), Docetaxel (01885), Doxorubicin (D1515), Paclitaxel (T7402) were from Sigma. Phenethyl isothiocyanate (PEITC, 253731) was from Aldrich. E-64-D (BML-PI107), Pepstatin A (ALX-260-085) were from Enzo Life Sciences. Z-VAD-FMK (A1902) was from APExBIO. Ferroquine and amino-ferrocene were provided by Prof. Christophe Biot, University Lille1, France.

### Constructs and transfection

The pDest-mCherry-eGFP-LC3B plasmid was kindly provided by Prof. Terje Johansen (Institute of Medical Biology, University of Tromso, Tromso, Norway)^47^. The pEGFP-LC3 (human) was a gift from Toren Finkel (Addgene plasmid #24920)^48^. LAMP1-mGFP was a gift from Esteban Dell’Angelica (Addgene plasmid #34831)^49^. pAcGFP1-Mito was from Clontech.

Cells were transiently transfected with selected constructs using X-tremeGENE™ HP DNA Transfection Reagent (Roche Life Science) following the manufacturer’s instructions. In 24 h the cells were washed with fresh culture medium and left for another 24 h before treatments and/or confocal imaging.

### Cell culture

Normal prostate epithelial PNT1A cell line (from European Collection and Cell Culture (ECACC)), prostate cancer cell line PC3M (provided by Prof. M. Djamgoz, Imperial College London) as well as prostate cancer cell lines LNCaP, C4-2, PC3 and DU-145 from the American Type Culture Collection (ATCC) were cultured in RPMI 1640 medium (31870, Gibco-Life Technologies) supplemented with 5 mM L-glutamine (25030, Gibco) and 10% fetal bovine serum (FBS) (10270, Gibco). For serum-starvation conditions, cancer cells were cultured in medium without FBS and L-glutamine. For complete starvation conditions, cells were incubated in glucose-free HEPES-buffered salt solution (HBS) containing: 140 mM NaCl, 5 mM KCl, 2 mM CaCl_2_, 1 mM MgCl_2_, 10 mM HEPES at pH 7,4.

Pancreatic adenocarcinoma cell line Panc1 from the ATCC was cultured in Dulbecco’s minimal essential medium DMEM + GlutaMAX (31966, Invitrogen, Life Technologies Inc.) supplemented with 10% FCS (PAA Gold). Pancreatic adenocarcinoma cell line MiaPaca2 from the ATCC was cultured in DMEM/F12 medium (31330, Gibco-Life Technologies) supplemented with 2.5% Horse Serum (S9135, Biochrom) and 10% FCS (PAA Gold).

All cells were maintained in 5% CO_2_, 95% air at 37 °C in a humidified incubator. For hypoxic conditions cells were maintained in a Galaxy 170R CO_2_ incubator (New Brunswick, Eppendorf) in 5% CO_2_, 1.2% O_2_ and 93.8% N_2_ at 37 °C for 48 h.

### Cell treatments

Ferroquine was dissolved in DMSO to produce 10 mM stock solution. Chloroquine diphosphate was dissolved in ddH_2_O to get 10 mM stock solution. To compare effects of FQ and CQ, respective amounts of DMSO were added to all CQ-containing solutions. DMSO was also routinely added to control solutions. Cisplatin was used at 5 μM, docetaxel at 2.5 nM, doxorubicin at 25 nM, paclitaxel at 5 nM PEITC 10 μM, z-VAD-FMK was used at 20 μM, necrostatin-1 at 30 μM, pepstatin A and E64d at 10 μM, wortmannin at 100 nM, bafilomycin A1 at 50 nM, ferrostatin-1 at 20 μM, deferoxamine at 50 μM. In the experiments with cell death inhibitors, cells were first pretreated with different cell death inhibitors for 3 h and then FQ was added to the cells.

### Cell viability

Cells were seeded at 5000 cells/well on 96-well plates in complete medium. In 48 h cells were treated with different compounds for up to 72 h in complete or serum-starved media. Cell viability was monitored using the CellTiter 96 Aqueous One Solution cell proliferation assay (Promega), on the basis of the cellular conversion of the colorimetric reagent MTS [3,4-(5-dimethylthiazol-2-yl)-5-(3-carboxymethoxyphenyl)-2-(4-sulfophenyl)-2H-tetrazolium salt] into soluble formazan by dehydrogenase enzymes found only in metabolically active cells. Following treatment the cells were incubated with reagent solution and absorbance was recorded at 490 nm wavelength using TriStar^2^ Multimode Reader LB942 (Berthold Technologies).

### Cytotoxicity assay

Cytotoxicity has been assessed based on the measurement of activity of lactate dehydrogenase (LDH) released from damaged cells. LNCaP or PC3M cells were seeded at 5000 cells/well on 96-well plates in complete medium. In 48 h cells were treated with vehicle, FQ or CQ for 24 h and released LDH activity has been analyzed using Cytotoxicity detection kit (LDH) (11644793001, Roche) following manufacturer’s guidelines.

### Clonogenic assay

LNCaP or PC3M cells were seeded in 6-well plates at a density of 1000 cells per well. In 24 h cells were treated with vehicle, CQ (7 μM) or FQ (7 μM) for 24 h, washed with complete medium and left to recover for 10 days. Cells were then washed with PBS, fixed with methanol/acetic acid (3/1) for 10 min and stained with 0.5% crystal violet in methanol for 2 h at room temperature. Following multiple washes with tap water plates were left to air-dry for several hours at room temperature. Colonies were then photographed and colony area was analyzed using Fiji software.

### Cell cycle analysis

LNCaP and PC3M cells were seeded on 60 mm tissue culture dishes. Two days after plating cells were treated with vehicle, CQ (7 μM) or FQ (7 μM) for 72 h. Cells were then harvested by trypsinization, washed with PBS, and fixed with cold 70% ethanol for 1 h at −20 °C. After two washes with PBS cells were treated with RNAse A (50 μg/ml) for 15 min at 20 °C followed by incubation with propidium iodide (20 μg/ml) for 4 h at 20 °C. Cell cycle was analyzed using Cyan LX9 cytometer (Beckman Coulter). Data analysis was performed using Summit 4.3 software (Beckman Coulter).

### Nuclear morphology

Nuclear morphology was determined by Hoechst 33258 staining. LNCaP cells were plated on tissue culture dishes with cover glass bottom (FluoroDish, FD35, World Presicion Instruments, Inc.). In 48 h cells were treated with vehicle or FQ (20 μM) for 24 h. Nuclear morphology was assessed by confocal imaging.

### Tandem mCherry-eGFP reporter fluorescence assay

LNCaP cells were transiently transfected with mCherry-eGFP-LC3B construct using X-tremeGENE™ HP DNA Transfection Reagent (Roche Life Science) following the manufacturer’s instructions. In 24 h the cells were washed with fresh culture medium and left for another 24 h before treatments with vehicle, CQ or FQ followed by confocal imaging. Tandem mCherry-eGFP reporter fluorescence assay is based on the use of the pH-sensitive fluorescent tag consisting of a tandem fusion of the red, acid-insensitive mCherry and the acid-sensitive GFP. Under neutral pH both mCherry and GFP fluoresce and colocalize indicating an autophagosome which is not fused with acidic lysosome. Alternativelly, colocalization of mCherry and eGFP signals could point on an amphisome or autolysosome with decreased acidification and/or impaired proteolytic degradation. In contrast, mCherry signal without eGFP corresponds to an amphisome or autolysosome with physiologically acidic interior^47^.

### Confocal Microscopy

Live cell images were obtained using confocal laser scanning microscope (LSM 700, Carl Zeiss MicroImaging GmbH) with a Plan Apochromat 40×/1.3 numerical aperture oil immersion objective and equipped with a CO_2_ and thermocontrolled chamber. The images were analyzed in Zeiss LSM Image Browser software and prepared for publication using Fiji software^50,51^.

### Electron microscopy

Cell pellets were fixed with 2.5% glutaraldehyde in 0.1 M cacodylate buffer, pH 7.4 for at least 30 minutes at 4 °C. After fixation, the specimens were thoroughly washed in 0.1 M cacodylate buffer and then postfixed with 1% osmium tetroxide in the same buffer for 1 hour at room temperature, stained with 2% uranyl acetate in distilled water for 15 minutes, dehydrated in graded acetonitrile, and embedded in Epon. Ultrathin sections (80 to 90 nm thick) were cut on a Leica UC7, transferred on 150-mesh grids and contrasted with 2% uranyl acetate solution and Reynolds lead citrate solution. The electron micrographs were taken with a Hitachi H600 transmission electron microscope at 75 kV accelerating voltage.

### Immunoblot analysis

Cells were washed with cold PBS and lysed in ice-cold buffer containing: 50 mM Tris-HCl, 1% Triton X-100, 0.1% SDS, 150 mM NaCl, 2 mM EDTA, 0.5% Sodium deoxycholate, 50 mM NaF, a protease inhibitor cocktail (P8340, Sigma) and a phosphatase inhibitor cocktail PhosSTOP (Roche). The lysates were sonicated and centrifuged at 15,000 × g at 4 °C for 15 minutes to remove cell debris. Supernatant protein concentration was determined by the BCA protein assay kit (Pierce Biotechnology). 30 μg of total protein were subjected to SDS-PAGE followed by transfer to PVDF membranes using the Trans-Blot® SD semi-dry transfer cell (Bio-Rad). The membranes were blocked in a 5% fat-free milk containing TBST solution (20 mM Tris pH 7.5, 150 mM NaCl, and 0.1% Tween 20) for 1 h at room temperature. The membranes were next incubated overnight at 4 °C with primary antibodies, and then for 1 h at room temperature with secondary antibodies conjugated to horseradish peroxidase. After washing, the membranes were processed for chemiluminescence detection using SuperSignal West Dura Extended Duration Substrate (34076, Thermo Scientific) and analyzed using Amersham Imager 600 (GE Healthcare Life Sciences). Fiji software was employed for quantitative analysis.

### Immunostaining

LNCaP cells were grown on glass coverslips. Following treatments cells were rinsed with PBS, fixed with 4% paraformaldehyde for 10 min at room temperature and washed with PBS. Samples were permeabilized and blocked for 10 min in PBS containing 0.05% Tween 20, 1% BSA and 0.2 M glycine before incubation with primary antibodies overnight at 4 °C. Following three washes with PBS, cells were incubated with Alexa Fluor tagged secondary antibodies (Life Technologies) for 1 h at room temperature. Then cell DNA was stained using DAPI (D9542, Sigma) for 10 min at room temperature. After three washes with PBS the slides were mounted with Mowiol® on glass slides and subjected to subsequent confocal imaging.

### Lysosomal membrane permeabilization (LMP) assays

#### Acridine orange staining

LNCaP cells were seeded on tissue culture dishes with cover glass bottom (FluoroDish, FD35, World Presicion Instruments, Inc.). Two days after plating cells were incubated with acridine orange (AO) (1 μg/ml final concentration) for 15 min in 37 °C. Following two washes with culture medium cells were treated with vehicle, CQ (30 μM) or FQ (30 μM) for 1h and subjected to confocal imaging. Upon excitation by blue light AO emits at 525 nm (green). Due to its weak base properties AO accumulates in acidic organelles, such as lysosomes and autolysosomes, where it precipitates and emits at around 650 nm (red). Thus healthy acidic vesicles appear as red puncta in green cytoplasm. When the pH inside the acidic organelles increases, AO fluorescence switches from red to green. In the case of LMP, AO relocates from lysosomes to cytoplasm thus increasing cytoplasmic green fluorescence intensity.

#### Fluorescent dextran release assay

LNCaP cells were grown on tissue culture dishes with cover glass bottom (FluoroDish, FD35, World Presicion Instruments, Inc.). Two days after plating cells were incubated with FITC-dextran (10 kDa, 1 mg/ml) (FD10S, Sigma) for 7 h, washed and chased for 3 h in complete medium. Then cells were treated with vehicle, FQ (20 μM) or CQ (20 μM) for 12 h and dextran relocation was analyzed by confocal microscopy. In control non-treated cells FITC-dextran appears in puncta, consistent with its reported localization in lysosomes. LMP provokes release of FITC-dextran from lysosomes to cytosol thereby increasing cytoplasmic fluorescence intensity.

### Mitochondrial transmembrane potential

LNCaP or PC3M cells were seeded on tissue culture dishes with cover glass bottom (FluoroDish, FD35, World Presicion Instruments, Inc.). Changes in mitochondrial membrane potential (ΔΨ) were imaged by means of the fluorescent ΔΨ-dependent lipophilic cationic dye tetramethyl-rhodamine ethyl ester (TMRE) which accumulates in active mitochondria. LNCaP and PC3M cells were stained with TMRE (0.1 µM, 10 min), treated with vehicle, CQ (20 μM) or FQ (20 μM) for 2 h and changes in intensity of TMRE signal were analyzed by confocal microscopy. To confirm the mitochondrial origin of the TMRE signal, at the end of the imaging protocol the cells were exposed to 5 µmol/L FCCP. TMRE fluorescence was excited by the 543 nm line of a 5 mW HeNe ion laser and the emitted fluorescence signal was captured at wavelengths above 560 nm.

Alternatively, ΔΨ was assessed using another ΔΨ-sensitive probe 3,3′-Dihexyloxacarbocyanine Iodide (DiOC6(3)) (318426, Aldrich). LNCaP or PC3M cells were treated with vehicle, CQ (15 μM) or FQ (15 μM) for 15 h, stained with DiOC6(3) (40 nM) for 15 min at 37 °C and analyzed by flow cytometry using Cyan LX9 cytometer (Beckman Coulter). Data analysis was performed using Summit 4.3 software (Beckman Coulter).

### Animal studies

Studies involving animals, including housing and care, method of euthanasia, and experimental protocols were approved by the University of Lille Animal Care and Use Committee and were conducted in the animal house of the University of Lille in accordance with appropriate guidelines (C59-00913; protocol CEEA 202012). LNCaP cells (5 × 10^6^ cells/mouse) or PC3M cells (3 × 10^6^ cells/mouse) were injected subcutaneously in 50% (v:v) Matrigel (BD Biosciences) to 6- to 8-week-old female nude mice. After tumor manifestation, mice were treated with FQ (50 mg/kg) or vehicle alone daily for 5 days with 2 days off treatment for the total period of 22 days (PC3M) or 28 days (LNCaP). FQ was administered both orally and subcutaneously in close vicinity of tumors. For oral administration, FQ was suspended in PBS containing 8% (v/v) Tween 80 and 3% (v/v) Ethanol (final volume 100 μl/mouse) and homogenized using Precellys 24 homogenizer (Bertin Instruments). Suspension was administered to mice by oral gavage using plastic feeding tube (FTP-20-30, Phymep) attached to 1ml syringe. For subcutaneous injections, 100 μM FQ solution in PBS has been prepared (1% final DMSO). 100 μl of this solution (4.3 μg FQ per mouse, or around 0.2 mg/kg) was injected in close vicinity of tumors. Tumor size was measured using digital caliper, and tumor volume was calculated as volume = (length × width^2)/2. At the end of the study, tumors were extracted, weighted and homogenized in RIPA buffer using Precellys 24 homogenizer (Bertin Instruments) for protein extraction.

### Statistical analysis

Data were analyzed using Origin 7.0 (OriginLab). Statistical tests used for comparisons are indicated in each figure legends. Where two-sided t-test was employed, the normal distribution of data was verified using the Shapiro–Wilk test or the Kolmogorov-Smirnov test. Otherwise, Mann-Whitney test was employed for statistical analysis. Asterisks denote: *P < 0.05; **P < 0.01; ***P < 0.001.

### Data availability

All data generated or analyzed during this study are available from the authors upon reasonable request.

## Electronic supplementary material

Supplementary info
