# Supplementary material for: Ferroquine, the next generation antimalarial drug, has antitumor activity
Source: Sci Rep. 2017 Nov 21;7:15896. doi: 10.1038/s41598-017-16154-2 (PMC5698296; doi:10.1038/s41598-017-16154-2)

Supplementary Information

Ferroquine, the next generation antimalarial drug, has antitumor activity

Artem Kondratskyi[1*], Kateryna Kondratska[1], Fabien Vanden Abeele[1], Dmitri Gordienko[1], Charlotte Dubois[1,5], Robert-Allain Toillon[2], Christian Slomianny[1], Sébastien Lemière[3], Philippe Delcourt[1], Etienne Dewailly[1], Roman Skryma[1], Christophe Biot[4], and Natalia Prevarskaya[1*]

[1] Inserm, U-1003, Laboratory of Excellence, Ion Channels Science and Therapeutics, SIRIC ONCOLille, Université Lille 1, Villeneuve d'Ascq, France.

[2] Inserm U908, Université Lille 1, Villeneuve d'Ascq, France

[3] Univ. Lille Nord de France, EA 4515 - LGCgE - Université Lille 1, Cité scientifique, SN3, F-59655 Villeneuve d'Ascq, France

[4] Univ. Lille 1, UGSF, UMR 8576 CNRS, 59650 Villeneuve d'Ascq, France.

[5] Present address: Laboratory of Angiogenesis and Vascular Metabolism, Vesalius Research Center, Department of Oncology (KU Leuven) and Vesalius Research Center (VIB), Campus Gasthuisberg O&N4, Herestraat 49 - 912, B-3000, Leuven, Belgium.

*Correspondence to: Artem Kondratskyi E-mail: Artem.Kondratskyi@inserm.fr; and Natalia Prevarskaya; E-mail: Natacha.Prevarskaya@univ-lille1.fr.

Cell line	IC50 (24h)	IC50 (48h)	IC50 (72h)
**LNCaP**	11.6±1.8 (n=6)	6.3±1.3 (n=6)	4.7±0.9 (n=6)
**PC3M**	35.4±4.1 (n=3)	29.1±7.2 (n=3)	17.5±5.3 (n=3)
**C4-2**	19.4±2.8 (n=2)	8.9±0.79 (n=2)	7.1±1.4 (n=2)
**PC3**	24.1±13.6 (n=2)	12.9±4.1 (n=3)	5.8±3.1 (n=2)
**DU-145**	24.6±2.8 (n=2)	12.6±2.9 (n=2)	6.2±2.9 (n=2)
**MiaPaCa2**	26.2±4.5 (n=3)	15.7±4.3 (n=3)	15.4±3.2 (n=2)
**Panc1**	19.1±6.9 (n=2)	15.3±3.6 (n=2)	10.7±1.1 (n=2)

Supplementary table 1. FQ effectively reduces the viability of different cancer cell lines. LNCaP, PC3M, C4-2, PC3 and DU-145 prostate cancer cell lines as well as MiaPaCa2 and Panc1 pancreatic cancer cell lines were seeded at 5000 cells/well on 96-well plates in complete medium. In 48h cells were treated with FQ (different concentrations) for up to 72 h in complete media. Cell viability was monitored using the CellTiter 96 Aqueous One Solution cell proliferation assay (Promega) and $TriStar^2$ Multimode Reader LB942 (Berthold Technologies).

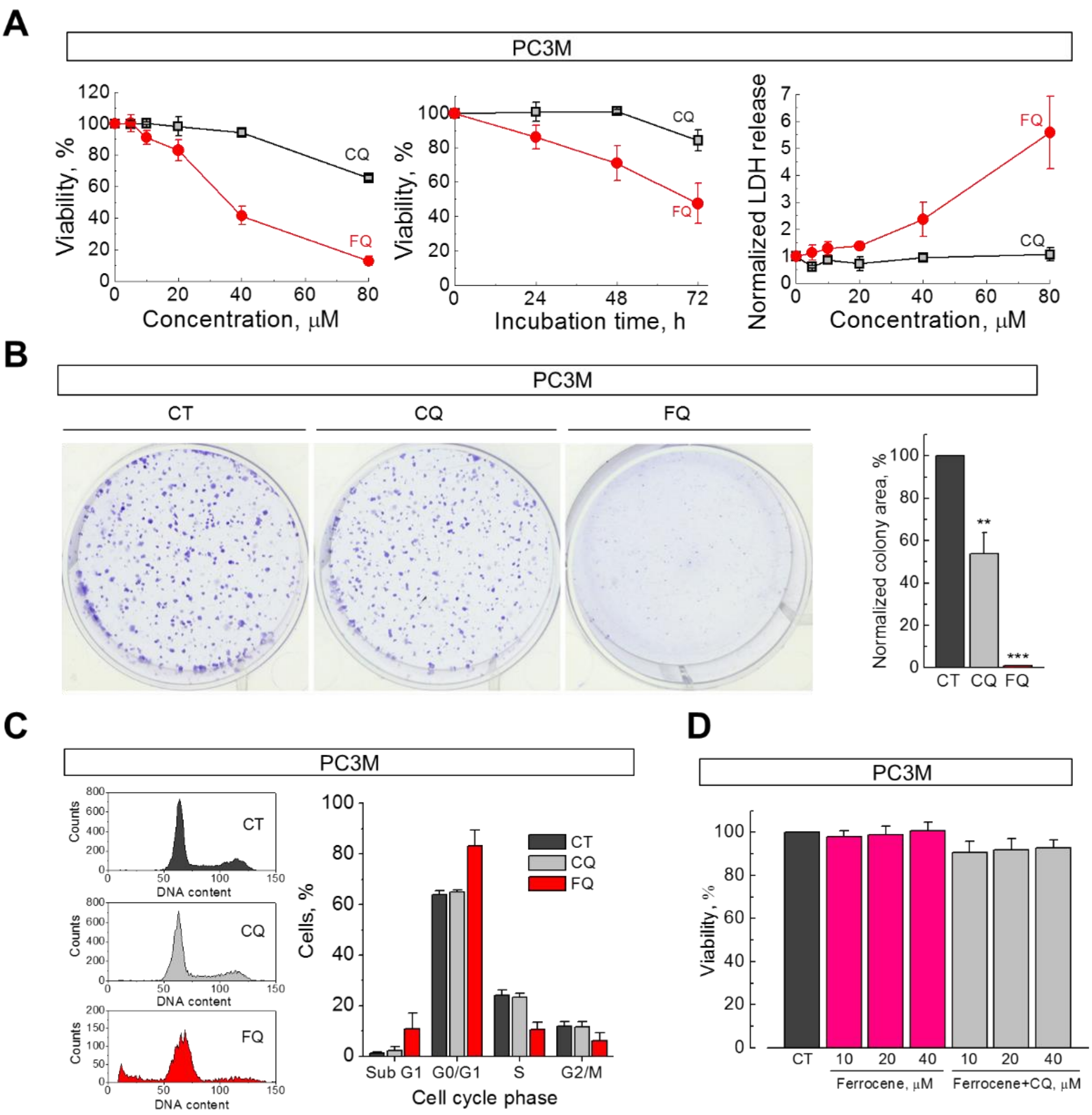

Supplementary Figure S1. Ferroquine induces proliferative arrest and death of PC3M prostate cancer cells. (A) Dose-response of FQ and CQ in viability (MTS, n=6) and cytotoxicity (LDH, n=3) assays on PC3M cells (24h). **(B)** Clonogenic survival of PC3M cells following treatment with vehicle, CQ (7μM) or FQ (7μM) for 24h (n=5); Mean ± SEM; paired t-test; **P<0.01; ***P<0.001. **(C)** Cell cycle analysis in PC3M cells treated with vehicle, CQ (7μM) or FQ (7μM) for 72h (n=3). **(D)** Effect of ferrocene and ferrocene+CQ on PC3M cell viability (48h, MTS, n=3).

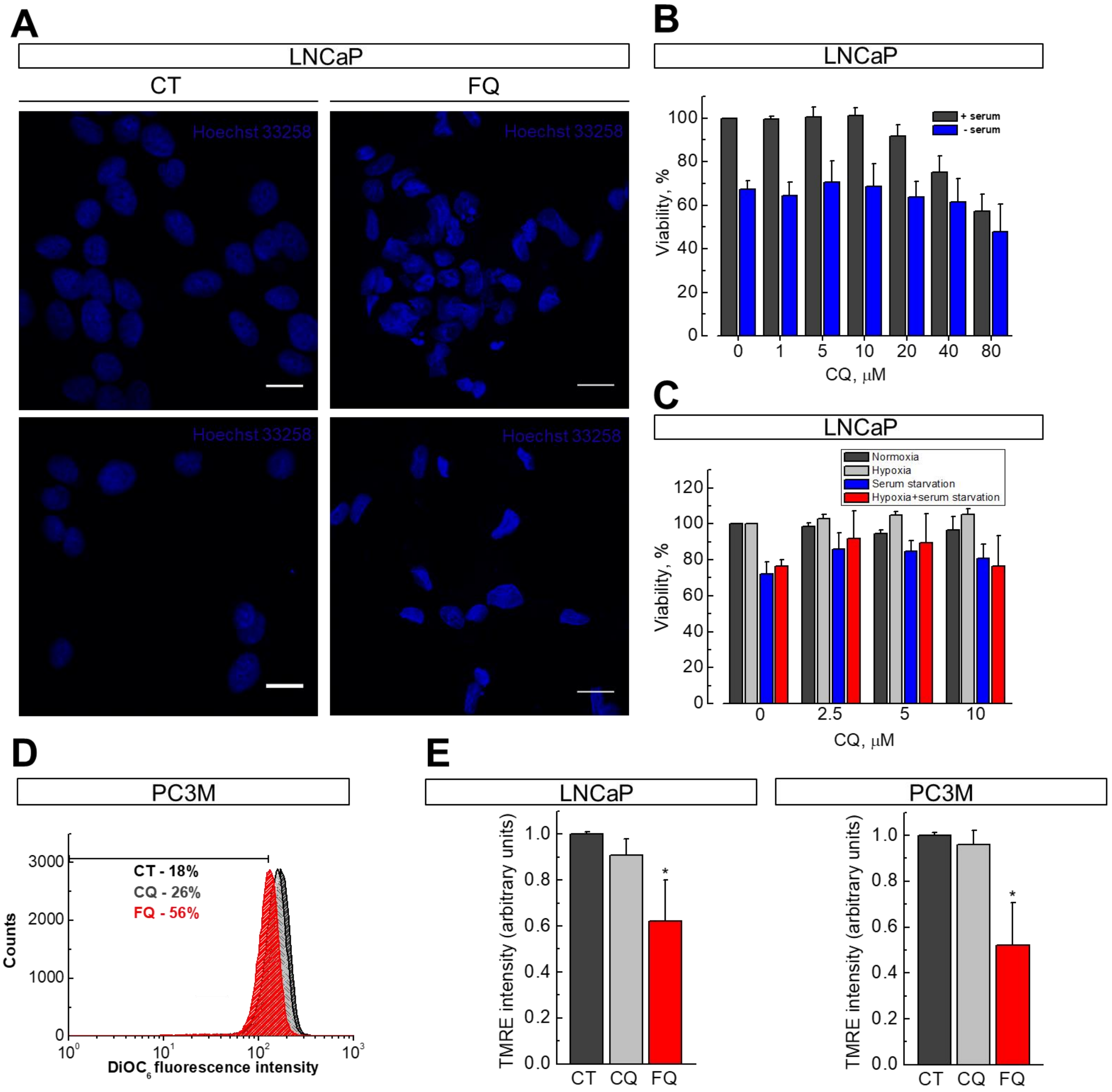

Supplementary Figure S2. FQ but not CQ sensitizes LNCaP cells to stress. (A) FQ alters nuclear morphology. LNCaP cell nuclear morphology following treatment with vehicle or FQ (20μM) for 24h as revealed by Hoechst 33258 staining. Scale bars, 20μm. **(B)** Effect of CQ on LNCaP cell viability in complete or serum-starved media (MTS, n=3). Mean ± SEM. **(C)** Effect of CQ on LNCaP cell viability in normoxic and hypoxic conditions (MTS, n=4). Mean ± SEM. **(D)** Flow cytometry experiment demonstrating increase in number of depolarized mitochondria induced by FQ (15μM, 15h) in PC3M cells (n=2). **(E)** FQ induces mitochondrial depolarization as revealed by TMRE staining of LNCaP and PC3M cells treated with vehicle (n=38 cells for LNCaP; n=31 for PC3M), CQ (20μM; n=34 cells for LNCaP; n=34 for PC3M), or FQ (20μM; n=49 cells for LNCaP; n=39 for PC3M). Mean ± SEM; t-test; *P<0.05.

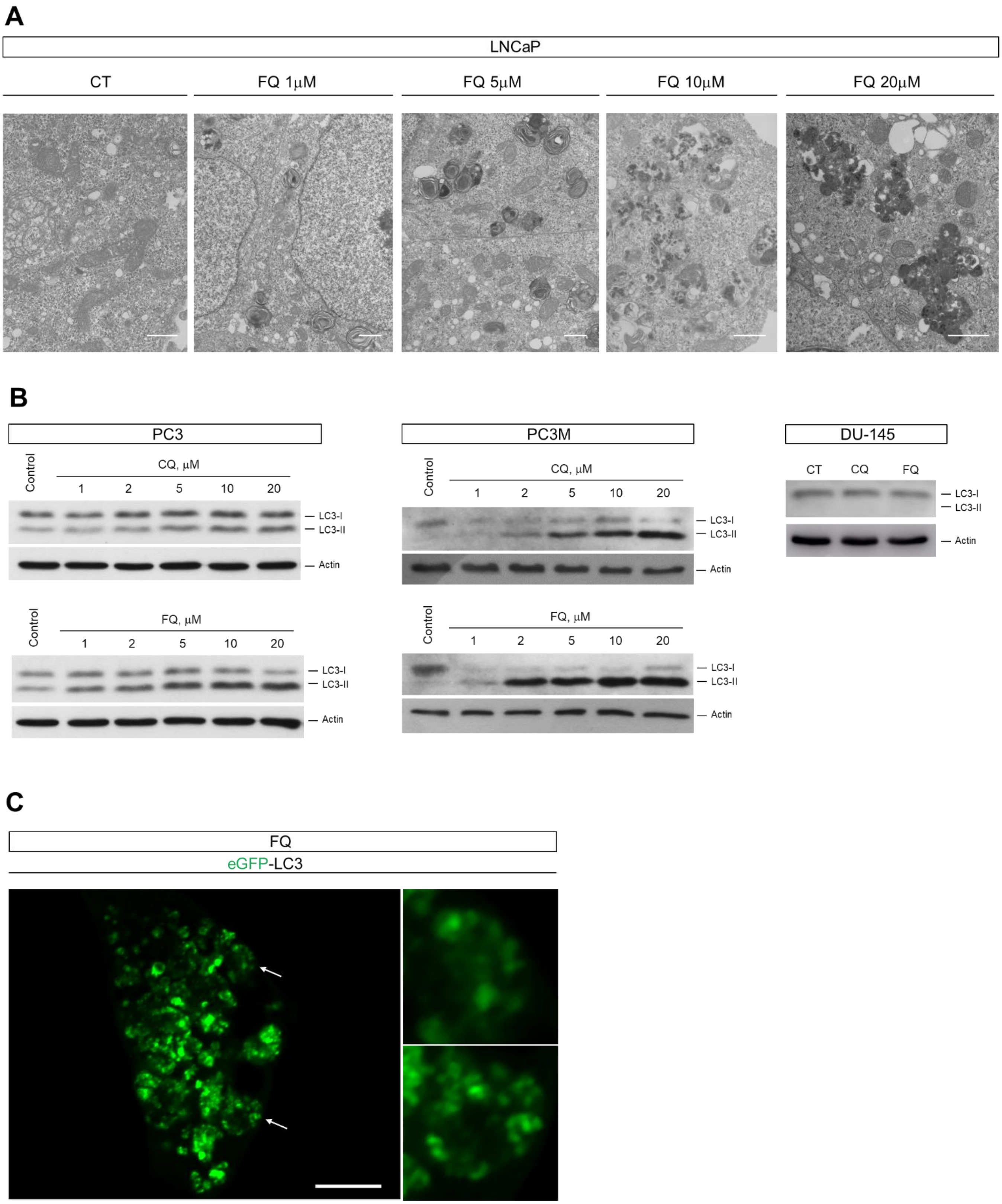

Supplementary Figure S3. Ferroquine induces accumulation of autophagic vacuoles. (A) Transmission electron microscopy images of LNCaP cells treated with vehicle or FQ (1, 5, 10 and 20μM) for 24h. Scale bar, 1μm. **(B)** Immunoblotting for LC3 and Actin in PC3, PC3M and DU-145 cells. Cells were treated with CQ or FQ for 12h. **(C)** Representative confocal images of LNCaP cells transfected with eGFP-LC3 and treated with FQ (10μM) for 24h. Scale bar, 10μm. Aggregates of puncta are shown.

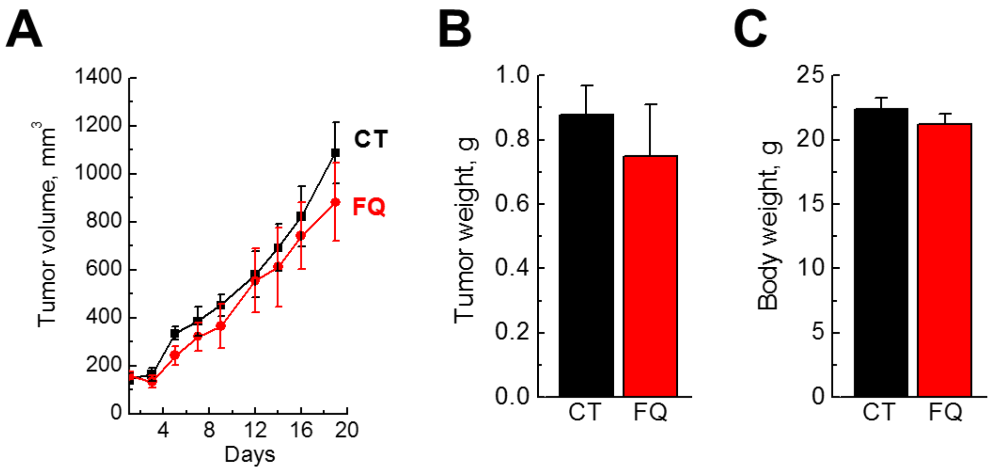

Supplementary Figure S4. Effect of FQ on PC3M cell-derived tumor xenograft growth *in vivo*. **(A)** *In vivo* antitumor effect of FQ (n=5). Data presented as Mean ± SEM. **(B)** Effect of FQ on tumor weight. Data presented as Mean ± SEM. **(C)** Effect of FQ on mice body weight. Data presented as Mean ± SEM.

Supplementary Figure S9. Full-length blots used in the main figures.

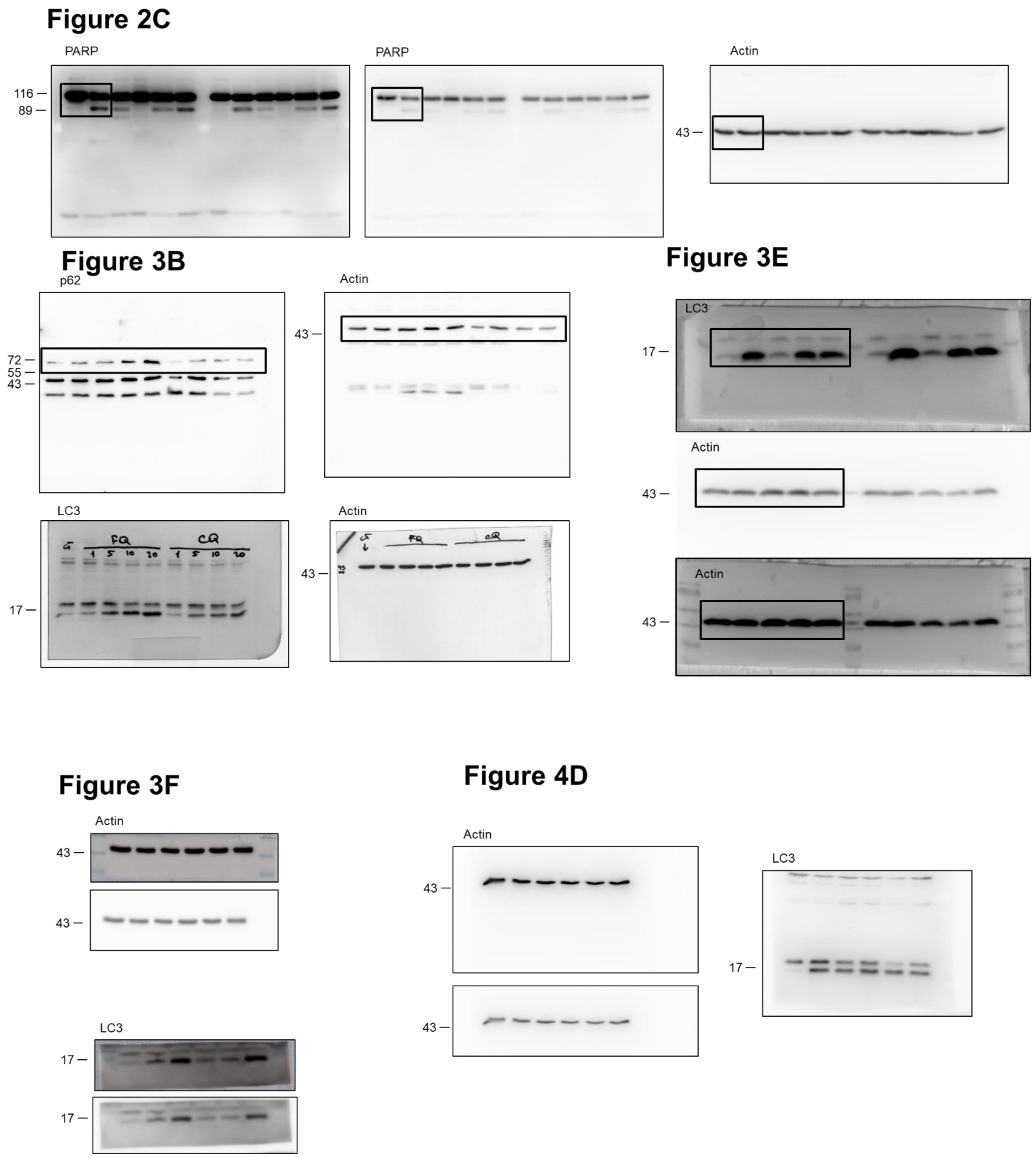

Supplement: Supplementary file 1 — Supplementary info [file 41598_2017_16154_MOESM1_ESM.pdf]
